# The difference in mean arterial pressure induced by remimazolam compared to etomidate in the presence of fentanyl at tracheal intubation: A randomized controlled trial

**DOI:** 10.3389/fphar.2023.1143784

**Published:** 2023-03-20

**Authors:** Xiaofang Huang, Huiyu Cao, Cuiwen Zhang, Hongmeng Lan, Xiaofang Gong, Ruijie Li, Yan Lin, Bing Xu, Huihe Chen, Xuehai Guan

**Affiliations:** ^1^ Department of Anesthesiology, the First Affiliated Hospital of Guangxi Medical University, Nanning, Guangxi, China; ^2^ Department of Rehabilitation, the People’s Hospital of Guangxi Zhuang Autonomous Region, Nanning, Guangxi, China; ^3^ Department of Rehabilitation, the First Affiliated Hospital of Guangxi Medical University, Nanning, Guangxi, China

**Keywords:** remimazolam, etomidate, post-induction hypotension, mean arterial pressure, hypnotic

## Abstract

**Background:** Combined use of hypnotic and opioids during anesthesia inductions decreases blood pressure. Post-induction hypotension (PIHO) is the most common side effect of anesthesia induction. We aimed to compare the difference in mean arterial pressure (MAP) induced by remimazolam with that induced by etomidate in the presence of fentanyl at tracheal intubation.

**Methods:** We assessed 138 adult patients with American Society of Anesthesiologists physical status I–II who underwent elective urological surgery. Patients were randomly allocated to receive either remimazolam or etomidate as alterative hypnotic in the presence of fentanyl during anesthesia induction. Comparable BIS values were achieved in both groups. The primary outcome was the difference in the MAP at tracheal intubation. The secondary outcomes included the characteristics of anesthesia, surgery, and adverse effects.

**Results:** The MAP was higher in the etomidate group than in the remimazolam group at tracheal intubation (108 [22] mmHg vs. 83 [16] mmHg; mean difference, −26; 95% confidence interval [CI], −33 to −19; *p* < 0.0001). Heart rate was significantly higher in the etomidate group than in the remimazolam group at tracheal intubation. The patients’ condition warranted the administration of ephedrine more frequently in the remimazolam group (22%) than in the etomidate group (5%) (*p* = 0.0042) during anesthesia induction. The remimazolam group had a lower incidence of hypertension (0% vs. 9%, *p* = 0.0133), myoclonus (0% vs. 47%, *p* < 0.001), and tachycardia (16% vs. 35%, *p* = 0.0148), and a higher incidence of PIHO (42% vs. 5%, *p* = 0.001) than the etomidate group during anesthesia induction.

**Conclusion:** Remimazolam was associated with lower MAP and lower heart rate compared to etomidate in the presence of fentanyl at tracheal intubation. Patients in the remimazolam group had a higher incidence of PIHO, and their condition warranted the administration of ephedrine more frequently than in the etomidate group during anesthesia induction.

## 1 Introduction

Combined use of hypnotic and opioids during anesthesia inductions decreases blood pressure. Intraoperative hypotension is associated with perioperative cardiovascular events (Walsh et al., 2013), acute kidney injury (Maheshwari et al., 2018), and neurocognitive disorders (Bijker et al., 2012; Krzych et al., 2020). Post-induction hypotension (PIHO) is a type of intraoperative hypotension that occurs in up to 60% of patients (Green and Butler, 2016). It is difficult to distinguish the adverse outcomes of PIHO from intraoperative hypotension throughout the operation. However, it is clear that the duration and severity of PIHO before incision are strongly associated with acute kidney injury (Maheshwari et al., 2018). Patients with PIHO have increased mortality (8.8%), intensive care unit length of stay (7.9%), and postoperative mechanical ventilation (20.7%) (Green and Butler, 2016).

PIHO is mainly due to cardiovascular depression associated with anesthetic drugs. Intravenous induction of anesthesia using etomidate, a hypnotic drug, is considered one of the most suitable methods for providing hemodynamic stability. However, shortcomings of etomidate, such as adrenocortical depression and myoclonus, have prevented its widespread use. The selection of an effective and safe hypnotic drug for general anesthesia is challenging.

Remimazolam is a novel ultra-short-acting benzodiazepine sedative that acts on γ-aminobutyric acid type A receptors (GABA_A_Rs). This drug differs from propofol owing to its low potential for respiratory and cardiovascular depression. Remimazolam has been considered for different conditions, such as endoscopic procedures, intensive care unit settings, and general anesthesia (Doi et al., 2020a; Doi et al., 2020b; Chen et al., 2021; Rex et al., 2021). The lack of hemodynamic depression is a good characteristic of remimazolam (Doi et al., 2020a; Doi et al., 2020b; Zhu et al., 2021), and consequently, its single use in anesthesia induction and maintenance has recently been licensed (China, November 2021; South Korea, January 2021; Japan, January 2020). Some case reports have revealed that remimazolam has been successfully used in general anesthesia (Nakayama et al., 2021).

No randomized controlled clinical trials have compared differences in cardiovascular depression between remimazolam and etomidate. In view of the growing evidence on mild cardiovascular depression with remimazolam, we aimed to compare the difference in mean arterial pressure (MAP) induced by remimazolam with that induced by etomidate at tracheal intubation. We hypothesized that in the presence of fentanyl, the MAP is lower in the remimazolam group than in the etomidate group at tracheal intubation.

## 2 Methods

### 2.1 Study design and patients

This prospective, single-center, single-blinded, and randomized clinical trial was approved by the Medical Ethics Committee of the First Affiliated Hospital of Guangxi Medical University (identifier: 2021-KY-E−276). The trial was registered before patient enrollment at Chinese Clinical Trial Registry (http://www.chictr.org.cn/showproj.aspx?proj=137224; registration NO; ChiCTR2100053118; principal investigator: Xuehai Guan; date of registration: 12 November 2021) and was performed at the First Affiliated Hospital of Guangxi Medical University. All patients in the trial were informed of the purpose of the study, and they all provided written informed consent. This trial was performed in accordance with the Declaration of Helsinki and adhered to CONSORT guidelines.

One hundred and thirty-eight patients with American Society of Anesthesiologists (ASA) physical status I–II, aged between 18 and 60 years, competent enough to provide informed consent, and scheduled for elective surgery in urology under general anesthesia, were enrolled in this study. Patients with a history of severe hypertension (systolic blood pressure ≥ 180 mmHg or diastolic blood pressure ≥ 110 mmHg), severe heart (NYHA class III or IV); difficult airway (Mallampati class III or IV); chronic obstructive pulmonary disease or asthma; adrenocortical dysfunction (serum cortisol < 3 μg/dL); liver dysfunction (Child-Pugh class B or C); or chronic kidney diseases (glomerular filtration rate < 60 mL/min); severe anemia (hemoglobin < 60 g/L); shock; hyperthyroidism, hypothyroidism or gout caused by abnormal purine metabolism; electrolyte disorders; alcoholism and drug addiction; delayed gastric retention or emptying; and abuse of hormones or narcotic analgesics were excluded. Further exclusion criteria were pregnancy, allergy to the study drugs, and surgical procedures requiring controlled hypotension.

### 2.2 Randomization and masking

Random numbers were generated using EpiCalc 2000 software. Patients were randomly allocated to one of two groups, and each group received either etomidate or remimazolam as hypnotic. Sealed envelopes were used to conceal group allocation. A few minutes before anesthesia induction, an assistant who did not participate in anesthesia opened the envelope to prepare the drugs. Patients, surgeons, attending anesthetists in charge of the post-anesthesia care unit (PACU), and data collectors were blinded to the grouping. Attending anesthetists performing the anesthesia were not blinded to group allocation. Group allocation was revealed only after data collection and analysis.

### 2.3 Intervention, anesthesia, and study outcomes

Patients fasted for 6 h. Only clear liquids were allowed for up to 2 h before anesthesia. No premedication was administered. A 20-gauge cannula was inserted into the vein on the dorsum of the hand and Ringer’s lactate was infused to maintain patency (5 mL/kg/h). The patients were monitored for non-invasive blood pressure (NIBP), five-lead electrocardiography, pulse oximetry (SpO_2_), and capnography by using an automatic monitor (C70, Comen, Shenzhen, China). The width of the cuff should cover 2/3 of the upper arm, and the length of the cuff should reach 2/3 of the circumference of the upper arm. NIBP was monitored by using a cyclic measuring way during anesthesia induction. During tracheal intubation, the cuff inflation started just with the beginning of intubation. The depth of anesthesia was monitored with a bispectral index (BIS; Covidien, MA, United States). Prior to the induction of anesthesia, 100% oxygen was administered for 3 min by placing a mask on the patient’s face. The doses and infusion rates of etomidate and remimazolam were based on drug instructions. In the etomidate group, anesthesia was induced using etomidate (Jiangsu Nhwa Pharmaceutical Co., Xuzhou, China) at 0.25 mg/kg/min until the BIS reached a range of 40–60. In the remimazolam group, anesthesia was induced using remimazolam tosilate (Jiangsu Hengrui Medicine Co., Lianyungang, China; diluted with normal saline to 1 mg/mL), starting at 10 mg/kg/h until the BIS reached a range of 40–60 and was maintained between 0.2–2 mg/kg/h (Doi et al., 2020a; Sheng et al., 2020). After loss of responsiveness (LOR, Modified Observer’s Assessment of Alertness/Sedation = 0, performed with 15 s intervals), cisatracurium (0.3 mg/kg IV; Sinopharm Chemical Reagent Co., Shanghai, China) and fentanyl (3 ug/kg IV; Yichang Humanwell Pharmaceutical Co., Yichang, China) was administered. The patients were intubated 3 min after cisatracurium administration. Remimazolam and etomidate were administered in a near-equipotent fashion by maintaining the BIS value between 40 and 60 from induction to 5 min after intubation. After stopping etomidate or remimazolam, anesthesia was maintained using a combination of propofol (Guangdong JiaBo Pharmaceutical Co., Qingyuan, China) and remifentanil (Yichang Humanwell Pharmaceutical Co., Yichang, China) using a target-controlled infusion pump in both groups. The infusion rates of propofol (plasma concentration: 2.5–4 μg/mL) and remifentanil (plasma concentration: 2.5–4 ng/mL) were regulated according to clinical signs and symptoms (changes in blood pressure and heart rate, tears, sweating, etc.), the BIS value (maintained at a range of 40–60), and the overall condition of the patients. Cisatracurium was administered as a repeated bolus dose of 0.1–0.2 mg/kg. All patients were mechanically ventilated (tidal volume, 8 mL/kg; respiratory rate: 12 breaths/min; oxygen concentration: 60%; fresh gas flow: 2 L/min). Attending anesthetists adjusted for the drug and fluid administration. All episodes of hypotension (30% decrease in MAP from baseline) were treated with ephedrine boluses (5 mg) at the discretion of the anesthesia provider, and no other vasopressors were used during induction.

If signs of intraoperative awakening were detected, the infusion rate of propofol was adjusted to 10 mL/min for up to 1 min. If signs of awakening persisted, propofol was discontinued and replaced with sevoflurane.

All surgeries were performed according to clinical practice, and all drugs were discontinued at the end of surgery. All patients were transferred to the PACU for recovery. Muscle relaxant antagonists (neostigmine) were administered, if necessary.

### 2.4 Outcome measures

The primary outcome was the difference in MAP at tracheal intubation.

The secondary outcomes included vital signs, characteristics of anesthesia and surgery, and adverse effects during general anesthesia induction. NIBP was recorded at the following time points: 5 min before anesthesia (baseline), at LOR, at BIS ≤ 60, at intubation, and at 1 and 5 min after intubation. Heart rate, SpO_2_, and BIS values were recorded at the following time points: 5 min before anesthesia, at LOR, at BIS ≤ 60, at intubation, and at 1 and 5 min after intubation. Respiratory rates were recorded 5 min before anesthesia, at LOR, and at BIS ≤ 60. The time from the onset of anesthesia induction to LOR and from the induction of anesthesia to BIS ≤ 60 were calculated. The administration of etomidate, remimazolam, and vasoactive drugs was recorded from the induction of anesthesia to LOR, at BIS ≤ 60, and at tracheal intubation. Numbers of ephedrine administration during induction, propofol administration, remifentanil administration and total crystalloid infusion volume were also recorded. Adverse effects such as injection pain, PIHO (≥30% decrease in MAP from baseline from the beginning of anesthesia induction to 5 min after intubation), hypertension (≥30% increase in MAP from baseline), shedding tears, myoclonus, hiccups, and awareness were recorded during anesthesia induction (defined from induction to 5 min after intubation). Bradycardia (<50 beats/min) and tachycardia (>100 beats/min) were recorded from the beginning of anesthesia to the end of surgery. Dysphoria, postoperative delirium, and postoperative nausea/vomiting (PONV) were recorded from the ending of surgery to 24 h after surgery.

### 2.5 Sample size calculation

The sample size was calculated using PASS (version 11.0; NCSS Statistical Software, Utah, United States). Our preliminary experiments showed that the MAP (means [standard deviation, SD]) was 84.0 (12) mmHg and 94.0 (24) mmHg at intubation under anesthesia with remimazolam and etomidate, respectively (unpublished). This calculation assumed a type-I error of 0.05. Based on this, 58 samples were required for each group (two-sample *t*-test power analysis; two-sided alpha = 5%, power = 80%). Considering the potential ineligibility and loss to follow-up, we increased the total sample size to 138 by 20%.

### 2.6 Statistical analysis

Continuous variables were initially tested for normality using a Kolmogorov–Smirnov test, and subsequently tested for equality of variances using a F test. Values with normal distribution and equal variance are expressed as means (SD), and values with non-normal distribution or with unequal variance are reported as medians (interquartile range [IQR]). Categorical variables are expressed as numbers (proportions). Absolute standardized difference (ASD) was determined to identify any imbalance in baseline characteristics, and an ASD value > 0.34 was regarded as an imbalance with a limited sample size (Austin, 2009). Serial continuous data, such as NIBP, heart rate, SpO_2_ and categorical data BIS were compared using repeated-measures two-way analysis of variance (ANOVA) with Geisser–Greenhouse correction, followed by Bonferroni’s multiple comparison test. The baseline value of the outcomes was included as a covariate. The fixed effects were the group, time, and group × time interactions. Matching effects were used for the study patients. For a statistically significant interaction (*p* < 0.05), the data for the two groups were compared at each time point using Bonferroni’s multiple comparisons test (alpha = 0.05, with *p* values adjusted for multiple comparisons and significant if *p* < .05). For a statistically non-significant interaction (*p* > 0.05), the main effect of group was reported. Variables with non-normally distributed data, such as the characteristic of anesthesia and surgery, the administration of hypnotic, remifentanil, ephedrine and crystalloid infusion volume, were reported as median (IQR) and analyzed using the Mann–Whitney *U* test between the two groups. Other continuous data were compared using an unpaired *t*-test where appropriate. Categorical data, including incidence of PIHO, injection pain, hypertension, bradycardia, tachycardia, dysphoria, hiccups, delirium, PONV, shedding tears, myoclonus, awareness and numbers of using ephedrine were analyzed using Chi-square test (when < 20% cells have an expected count less than 5) or Fisher’s test (when ≥ 20% cells have an expected count less than 5, or at least one cell has an expected count less than 1) followed by Koopman asymptotic score, as appropriate. Statistical analyses were performed using GraphPad Prism 9.0 (Dotmatics, Boston, USA). A *p*-value of < 0.05 was considered statistically significant.

## 3 Results

**FIGURE 1:**
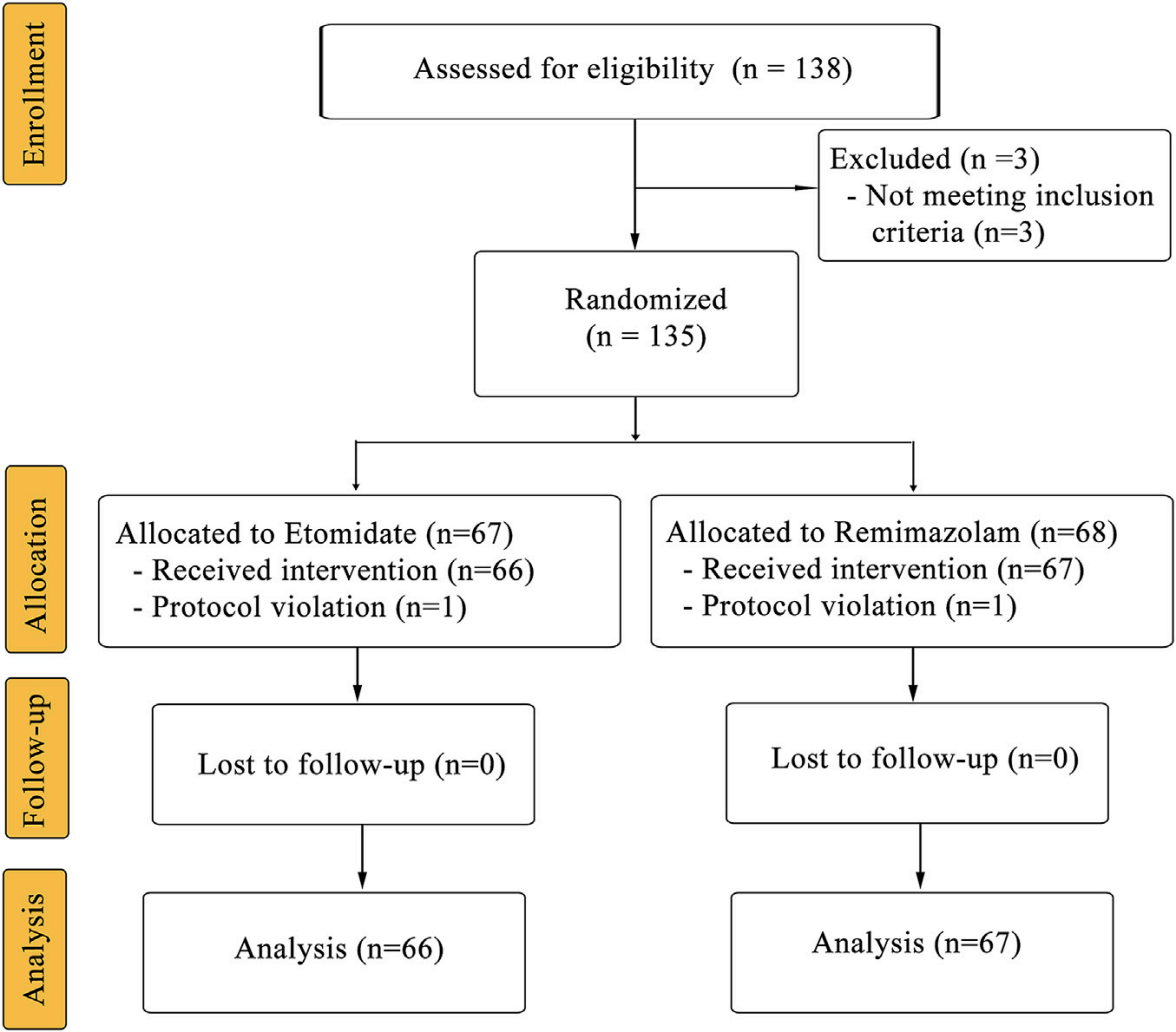
CONSORT diagram for the trial. CONSORT indicates Consolidated Standards for Reporting of Trials.

One hundred and thirty-eight patients were assessed for eligibility (Figure 1; 15 November 2021–31 March 2022). Five patients were excluded from the analysis due to failure to meet the inclusion criteria (*n* = 3) or protocol violation (*n* = 2). Patient characteristics did not differ between the groups in terms of age, height, body weight, body mass index (BMI), sex, or ASA physical status (Table 1).

**TABLE 1:** Demographic data in patients receiving etomidate or remimazolam for induction of anesthesia.

Characteristics	Etomidate (*n* = 66)	Remimazolam (*n* = 67)	ASD	*p-*value
Age (y)	46 (10)	43 (10)	0.33	0.0512^a^
Height (cm)	164 (7)	165 (7)	0.22	0.2070^a^
Body weight (kg)	65 (11)	68 (13)	0.25	0.1501^a^
BMI (kg/m^2^)	24 (3)	25 (3)	0.16	0.3765^a^
Male (%)	44 (67%)	48 (72%)	0.13	0.7059^b^
ASA physical status (%)			0.32	0.1276^c^
I	3 (5%)	9 (13%)		
II	63 (95%)	58 (87%)		

Data are displayed means (standard deviation) or numbers (proportions). 1.96 * √ ((n1 + n2)/n1n2) = 0.34, and all the ASD, in the table is smaller than 0.34.

^a^ Obtained from a unpaired *t*-test.

^b^ Obtained from a Chi-square test.

^c^ Obtained from Fisher’s exact test. Abbreviation: BMI, body mass index; ASA, American Society of Anesthesiologists; ASD, absolute standardized difference.

The primary outcomes are presented in Table 2. The MAP was higher in the etomidate group than in the remimazolam group at tracheal intubation (108 [22] mmHg vs. 83 [16] mmHg; mean difference, −26; 95% confidence interval [CI], −32 to −19; *p* < 0.0001).

**TABLE 2:** MAP in patients receiving etomidate or remimazolam for induction of anesthesia (mmHg).

Time point	Etomidate (*n* = 66)	Remimazolam (*n* = 67)	Effect size	*p*-value
5 min before anesthesia	102 (13)	102 (15)	−0.8 (−8 to 6)	> 0.9999
At LOR	98 (13)	91 (16)	−7.2 (−14 to −0.07)	0.0301
At BIS ≤ 60	95 (13)	86 (13)	−9 (−16 to −2)	0.0005
At intubation	108 (22)	83 (16)	−26 (−33 to −19)	< 0.0001
1 min after intubation	105 (19)	84 (16)	−21 (−28 to −14)	< 0.0001
5 min after intubation	93 (14)	75 (14)	−18 (−26 to −11)	< 0.0001

Data are displayed as means (standard deviation).

All *p* values were obtained from two-way repeated-measures analysis of variance, with effect size presented as difference in means (95% CI). Drug: F (1, 131) = 40.12, *p* < 0.0001; Time: F (5, 655) = 46.12, *p* < 0.0001; Drug ⅹ Time: F (5, 655) = 28.93, *p* < 0.0001; Subject: F (131, 655) = 9.00, *p* < 0.0001.

Abbreviation: MAP, mean arterial pressure; LOR, loss of responsiveness; BIS, bispectral index.

**FIGURE 2:**
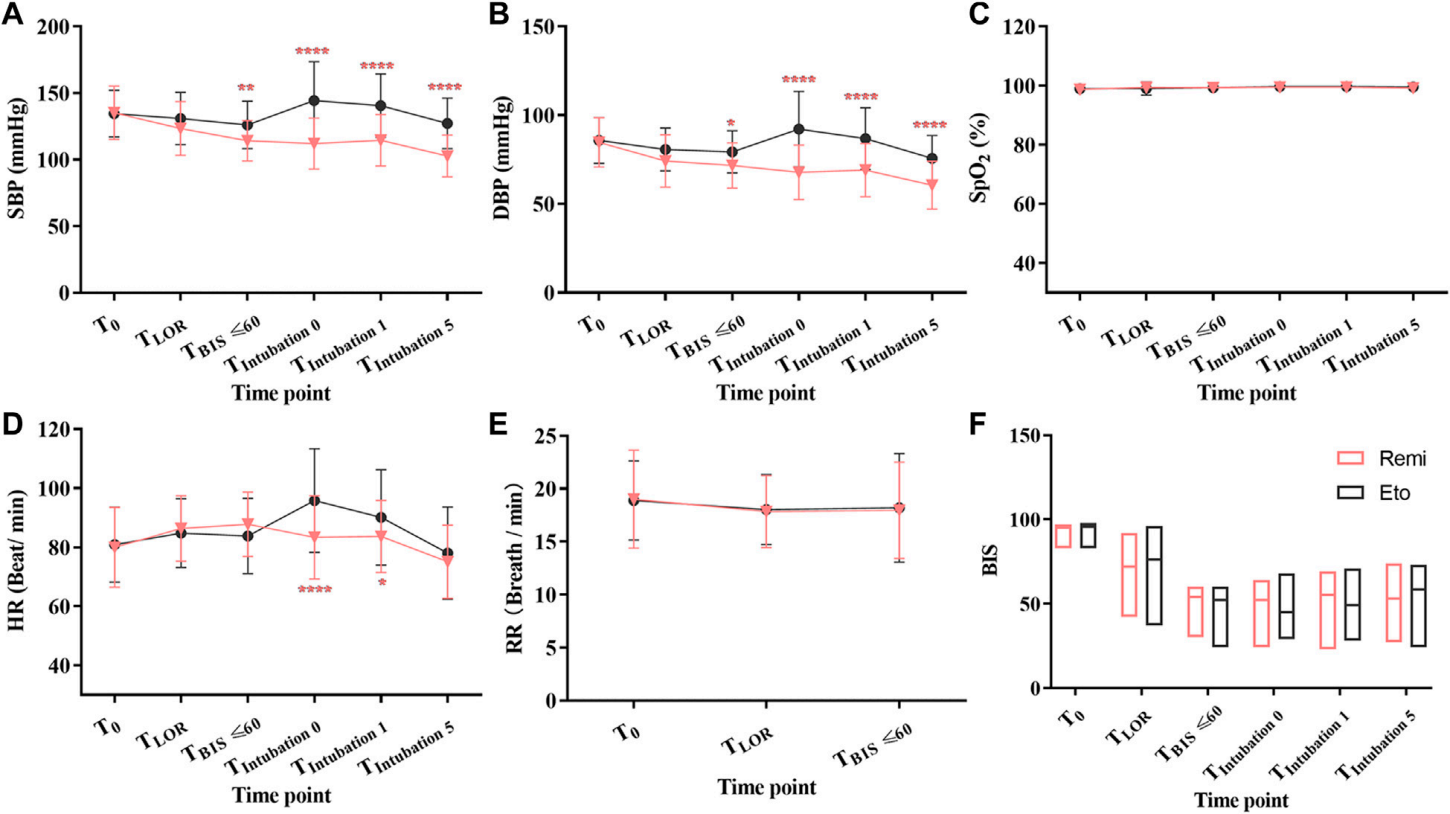
Changes of vital signs of patients receiving etomidate (circle) or remimazolam (inverted triangle) for induction of anesthesia. Data are displayed as means (SD) **(A–E)** or medians (IQR) **(F)**. *p < 0.05, **p < 0.01, ****p < 0.0001, compared with remimazolam group. Data were compared using repeated-measures two-way analysis of variance (ANOVA) with Geisser-Greenhouse correction, followed by Bonferroni’s multiple comparisons test. Abbreviations: Eto, etomidate; Remi: remimazolam; SBP, systolic blood pressure; DBP, diastolic blood pressure; HR, heart rate; SpO_2_, pulse oximetry; RR: respiratory rate; LOR: loss of responsiveness; BIS, bispectral index.

The MAP was higher in the etomidate group than in the remimazolam group at other time points during anesthesia induction (at LOR: 98 [13] mmHg vs. 91 [16] mmHg, mean difference, −7; 95% CI, −14 to −0.07; *p* = 0.0301; at BIS ≤ 60: 95 [13] mmHg vs. 86 [13] mmHg, mean difference, −9; 95% CI, −16 to −2; *p* = 0.0005; at 1 min after intubation: 105 [19] mmHg vs. 84 [16] mmHg, mean difference, −21; 95% CI, −28 to −14; *p* < 0.0001)); at 5 min after intubation, 93 [14] mmHg vs. 75 [14] mmHg, mean difference, −18; 95% CI, −26 to −11, *p* < 0.0001) (Table 2). Systolic blood pressure (SBP) and diastolic blood pressure (DBP) were higher in the etomidate group than in the remimazolam group at BIS ≤ 60, at tracheal intubation, at 1 min and 5 min after tracheal intubation (Figures 2A, B). The heart rate was higher in the etomidate group compared to the remimazolam group at tracheal intubation (*p* < 0.0001) and 1 min after tracheal intubation (*p* < 0.05) (Figure 2D). There was no significant difference in respiratory rate, SpO_2_, or BIS between the two groups at the time points we designed (Figures 2C, E, F).

The characteristics of the anesthesia are shown in Table 3. The time from induction to LOR was shorter in the etomidate group (53 [40–66] s) than in the remimazolam group (69 [59–81] s) (*p* < 0.0001). The time from induction to BIS ≤ 60 was shorter in the etomidate group 85 [67–105] s) than in the remimazolam group (89 [77–124] s) (*p* = 0.1734). The patients’ condition warranted the administration of ephedrine more frequently in the remimazolam group (22%) than in the etomidate group (5%) (*p* = 0.0042) during anesthesia induction. The administration of propofol during anesthesia maintenance was lower in the remimazolam group than in the etomidate group (*p* = 0.0009). There was no significant difference in duration of anesthesia, duration of surgery, administration of remifentanil and crystalloid infusion between groups (*p* > 0.05).

**TABLE 3:** Characteristic of anesthesia and surgery in patients receiving etomidate or remimazolam for induction of anesthesia.

Outcomes	Etomidate (*n* = 66)	Remimazolam (*n* = 67)	Effect size	*p*-value
Time from anesthesia induction to LOR (s)	53 (40–66)	69 (59–81)	16 (10–23)	< 0.0001
Time from anesthesia induction to BIS ≤ 60 (s)	85 (67–105)	89 (77–124)	4 (−3–20)	0.1734
Total administration of hypnotic drugs during induction (mg)	20 (20–20)	36 (30–36)	16 (11–16)	< 0.0001
At LOR (mg)	14 (12–17)	22 (20–26)	8 (6–10)	< 0.0001
At BIS ≤ 60 (mg)	20 (16–20)	26 (23–32)	6 (6–10)	< 0.0001
At intubation (mg)	20 (20–20)	36 (30–36)	16 (11–16)	< 0.0001
Numbers of ephedrine administration during induction (%)	3 (5%)	15 (22%)	0.20 (0.06–0.62)	0.0042^a^
Total administration of propofol (mg)	507 (357–668)	376 (167–531)	−131 (−195 to −47)	0.0009
Total administration of remifentanil (μg)	490 (355–633)	382 (254–648)	−108 (−155 to 7)	0.0741
Duration of anesthesia (min)	82 (65–109)	79 (61–107)	−3 (−11 to 11)	0.9830
Duration of surgery (min)	51 (38–88)	54 (37–79)	3.5 (−8–10)	0.8479
Crystalloid infusion volume (mL)	680 (600–889)	640 (540–855)	−40 (−100 to 5)	0.1026

Data are displayed as medians (IQR).

^a^ Obtained from a Fisher’s exact test, with effect size presented as relative risk (95% CI).

All other *p*-value were obtained from a Mann-Whitney *U* test, with effect size presented as difference in median (95% CI).

Abbreviations: LOR, loss of responsiveness; BIS, bispectral index.

All adverse events were classified as mild or moderate (Table 4). The most reported adverse events were PIHO, myoclonus, tachycardia, and hypertension. A significantly higher incidence of hypotension was observed in the remimazolam group (42%) than in the etomidate group (5%) during the induction period (*p* = 0.001). The incidence of myoclonus was particularly increased in the etomidate group (47%) than in the remimazolam group (0%) (*p* < 0.0001). The proportion of patients who developed tachycardia during anesthesia in the remimazolam group (16%) was lower than that in the etomidate group (35%) (*p* = 0.0148). No difference was found in the percentage of bradycardia in the remimazolam group (6%) and in the etomidate group (6%) (*p* > 0.9999). The proportion of patients who developed hypertension during anesthesia in the remimazolam group (0%) was lower than that in the etomidate group (9%) (*p* = 0.0133). There was no significant difference between the groups in the percentages of patients with injection pain, dysphoria, hiccups, and tear shedding. None of the patients in either group developed delirium, nausea, vomiting, or awareness symptoms. There were no serious adverse events, deaths, or discontinuations due to adverse events.

**TABLE 4:** Incidence of adverse event in patients receiving etomidate or remimazolam for induction of anesthesia.

Outcomes	Etomidate (*n* = 66)	Remimazolam (*n* = 67)	Effect size	*p*-value
Injection pain	1 (2%)	1 (2%)	1.0 (0.11–9.6)	> 0.9999^a^
PIHO	3 (5%)	28 (42%)	0.21 (0.08–0.56)	0.0010^a^
Hypertension	6 (9%)	0 (0%)	Infinity (1.6 to Infinity)	0.0133^a^
Tachycardia (>100 beats/min)	23 (35%)	11 (16%)	2 (1–4)	0.0148^b^
Bradycardia (<50 beats/min)	4 (6%)	4 (6%)	1 (0.29–3.6)	> 0.9999^a^
Dysphoria	0 (0%)	1 (2%)	0 (0–3.9)	> 0.9999^a^
Hiccups	1 (2%)	2 (3%)	0.51 (0.07–3.8)	> 0.9999^a^
Delirium	0 (0%)	0 (0%)	NA	NA
Nausea/vomiting	0 (0%)	0 (0%)	NA	NA
Tear shedding	0 (0%)	4 (6%)	0 (0–1)	0.1194^a^
Myoclonus	31 (47%)	0 (0%)	Infinity (2 to Infinity)	< 0.0001^a^

Data are displayed as numbers (proportions).

^a^ Obtained from a Fisher’s exact test, with effect size presented as relative risk (95% CI).

^b^ Obtained from a Chi-square test, with effect size presented as relative risk (95% CI).

Abbreviation: NA, not applicable; PIHO, post-induction hypotension.

## 4 Discussion

To the best of our knowledge, the study is the first randomized controlled trial to compare the difference in MAP induced by remimazolam and etomidate in the presence of fentanyl at tracheal intubation. The main finding of our study was that the MAP was higher in the etomidate group than in the remimazolam group in the presence of fentanyl at tracheal intubation (Table 2). Additionally, heart rate was higher in the etomidate group compared to the remimazolam group at tracheal intubation. Patients in the remimazolam group had a higher incidence of PIHO, and the patients’ condition warranted the administration of ephedrine more frequently than in the etomidate group during anesthesia induction. Our results confirm our hypothesis that the MAP induced by remimazolam is lower than that induced by etomidate in the presence of fentanyl at tracheal intubation.

The distribution of volume, dynamic adjustments of cardiac output, and perfusion pressure rely on the preservation of adequate intravascular volume and autonomic nervous control system (Frandsen et al., 2022). General anesthesia is associated with cardiovascular depression and disruption of the autonomic nervous control system by anesthetic agents, which leads to redistribution of intravascular volume, thus resulting in PIHO. Etomidate is considered to be one of the most suitable anesthetic induction drugs because of its reliable sedation and hemodynamic stability (Aggarwal et al., 2016; Mohr et al., 2020; Dai et al., 2021). The lack of hemodynamic depression is a good characteristic of remimazolam (Doi et al., 2020a; Doi et al., 2020b; Zhu et al., 2021). The inhibitory effects of remimazolam on circulation are less than that of propofol (Chen et al., 2020; Chen et al., 2021; Zhang et al., 2021). Both etomidate and remimazolam are beneficial in patients with unstable hemodynamics. However, in our study, SBP, DBP and MAP was higher in the etomidate group than in the remimazolam group during anesthesia induction (Figure 2; Table 2). At intubation and 1 min after intubation, heart rate was significantly higher in etomidate group compared to the remimazolam group during anesthesia induction (Figure 2). The main reason for this may be the different mechanisms they work. GABA_A_Rs is a pentameric assembly of subunits drawn from multiple classes (α1-6, β1-3, γ1-3, δ1, and ε1), and is distributed throughout the brain (Whiting et al., 1995; Pirker et al., 2000). Positive allosteric modulation of GABA_A_Rs activity by general anesthetics represents a mechanism for central nervous system depression. Most general anesthetics are non-selective allosteric agonists of GABA_A_Rs. The etomidate binding site is formed by β and α subunits (Chiara et al., 2012). The ability of etomidate to modulate and activate GABA_A_Rs is dependent upon the β2-or β3-, but not β1 subunit (Belelli et al., 1997; Hill-Venning et al., 1997; Sanna et al., 1997). The β3-containing GABA_A_Rs mediates anesthesia induced by etomidate (Belelli et al., 1997; Jurd et al., 2003; Reynolds et al., 2003; O'Meara et al., 2004; Liao et al., 2005; Butovas et al., 2010; Amlong et al., 2016), β2-containing GABA_A_Rs contribute to the sedation induced by etomidate (Reynolds et al., 2003), and α5-containing GABA_A_Rs mediates the amnestic effects of etomidate (Cheng et al., 2006). These findings provide evidence that etomidate acts on different GABA_A_Rs subtypes, located in different neuronal circuits, to generate the multiple components of the anesthetic state. The benzodiazepine binding site is formed by the α and γ subunits, different from the binding site of other general anesthetics (Ernst et al., 2003; Ogris et al., 2004). It has been identified the GABA_A_Rs subtypes mediating specific aspects of the pharmacological spectrum of benzodiazepine too. For instance, the sedative and anterograde amnesic actions of diazepam is mediated by α1-containing GABA_A_Rs (Rudolph et al., 1999; McKernan et al., 2000), the anxiolytic action of diazepam is mediated by α2-containing GABA_A_Rs (Löw et al., 2000), whereas the myorelaxant action of diazepam is mediated by α2-,α3-and α5-containing GABA_A_Rs (Crestani et al., 2001; Crestani et al., 2002). Remimazolam, one of benzodiazepine intravenous anesthetics, is a high-affinity ligand for the benzodiazepine site on the GABA_A_Rs, showing similar activity between GABAA receptor subtypes (α1β2γ2, α2β2γ2, α3β2γ2, and α5β2γ2 subtypes) (Kilpatrick et al., 2007). We infer the different binding site and selectivity between etomidate and remimazolam may result in different hemodynamic changes. Another reason may be that etomidate does not significantly inhibit sympathetic tone and preserves autonomic reflexes (Ebert et al., 1992). It also acts as an agonist at the α2-adrenoceptors, in particular the α2B-adrenoreceptor responsible for the baroreflex (Paris et al., 2003; Rudolph and Antkowiak, 2004). Anesthetic induction doses of etomidate preserved cardiac index, systemic vascular resistance, and myocardial oxygen supply-to-demand ratio (Gooding and Corssen, 1977; Colvin et al., 1979; Gooding et al., 1979; Larsen et al., 1988; Brussel et al., 1989; Moller Petrun and Kamenik, 2013; Zgola et al., 2014). On the contrary, sedative and anesthetic doses of intravenous benzodiazepine induce a reduction of cardiac output, decrease the systemic vascular resistance and as a result of reduction in arterial blood pressure (Samuelson et al., 1981; Ruff and Reves, 1990). Remimazolam decreased autonomic nervous activity during anesthesia induction too (Hasegawa et al., 2022). Opioids showed synergistic effects with remimazolam in sedation, which decreased autonomic nervous activity (Zhou et al., 2021). Athough statistically different, it is possible that the different changes in NIBP observed in our study were not clinically relevant because we did not observe any serious complications. The duration and severity of PIHO was strongly associated acute kidney injury (Maheshwari et al., 2018). Routine therapy for maintaining hemodynamic stability included the use of different vasoactive drugs. The patients’ condition warranted more frequent administration of ephedrine, less propofol in the remimazolam group than that in the etomidate group (Table 3). One reason for this may be that we treated PIHO in a timely and effective manner, and the period of PIHO was too short to cause serious complications. Based on these results, we speculate that etomidate may be more beneficial for patients than remimazolam in the presence of fentanyl during the induction of anesthesia.

The onset time for etomidate was shorter than that for remimazolam (Table 3); however, only one injection speed was set for remimazolam and etomidate, and we could not exclude the possibility that the onset time depended on the injection speed. The proportion of patients who developed tachycardia and hypertension during anesthesia was higher in the etomidate group than in the remimazolam group. The incidence of etomidate-induced myoclonus was 47% (Table 4 **)**, which is consistent with previous reports (Schwarzkopf et al., 2003; Prakash et al., 2021). Myoclonus is not only bothersome but also increases the risk of vitreous prolapse in patients with open globe injury, pulmonary aspiration, and regurgitation in non-fasted patients (Van Keulen and Burton, 2003; Hüter et al., 2007). None of the patients in the remimazolam group developed myoclonus in our study. But whether remimazolam is more suitable for anesthesia induction than etomidate in patients with open-globe injury and non-fasted needs further study. PONV is a common phenomenon associated with etomidate use. Propofol has direct anti-emetic properties and can reduce PONV (Borgeat et al., 1992). In our study, PONV did not occur in the etomidate and remimazolam groups; however, we cannot directly conclude that remimazolam has anti-emetic properties unless the anti-emetic effect of propofol is excluded. Dysphoria, hiccups, and delirium were infrequent events in both groups.

Our study had some limitations. First, the external validity of the trial was weakened by the inclusion criteria of the patients and the single-center, single-blinded design. The study only included patients who underwent elective urological surgery. Therefore, our results may not be generalizable to patients undergoing other surgeries. All patients come from China. Further studies are needed to confirm our findings in other populations. Second, we did not assess some parameters related to cardiac function such as cardiac output or ejection fraction. Third, since only one injection rate of remimazolam was set, we could not get the most reasonable dose-effect relationship. Fourth, BIS is one of the most popular methods for monitoring the depth of anesthesia; however, the significance of using BIS to study remimazolam is not clear. Remimazolam has been shown to suppress BIS (Zhou et al., 2020). BIS monitoring was appropriate for assessing awareness signs during anesthesia with remimazolam (Sheng et al., 2020). Therefore, remimazolam and etomidate were administered in a near-equipotent fashion, maintaining a BIS value between 40 and 60 from induction to 5 min after intubation. If BIS is suitable for monitoring the anesthesia depth of remimazolam, its specific value requires further clinical data verification. Moreover, we did not record the duration of PIHO; therefore, we could not calculate the area under the hypotension-time curve. A higher incidence of PIHO does not necessarily indicate worse outcomes.

## 5 Conclusion

In conclusion, the MAP and heart rate was lower in the remimazolam group compared to the etomidate group in the presence of fentanyl at tracheal intubation. The incidence of PIHO was higher, and the patients’ condition warranted the administration of ephedrine more frequently in the remimazolam group compared to the etomidate group during anesthesia induction.

## Data Availability

The raw data supporting the conclusion of this article will be made available by the authors, without undue reservation.
